# Supplementary material for: Dynamic semantic cognition: Characterising coherent and controlled conceptual retrieval through time using magnetoencephalography and chronometric transcranial magnetic stimulation
Source: Cortex. 2018 Jun;103:329–49. doi: 10.1016/j.cortex.2018.03.024 (PMC6002612; doi:10.1016/j.cortex.2018.03.024)
Supplement: mmc1 [file mmc1.doc]

### Supplementary Materials

Supplementary Analysis 1: Whole-brain beamforming for a range of frequency bands

The most extensive changes in total power in response to the task were power decreases in the 25-35Hz frequency band. Data for all frequency bands from 200-400ms are provided in Supplementary Figure 1 below.


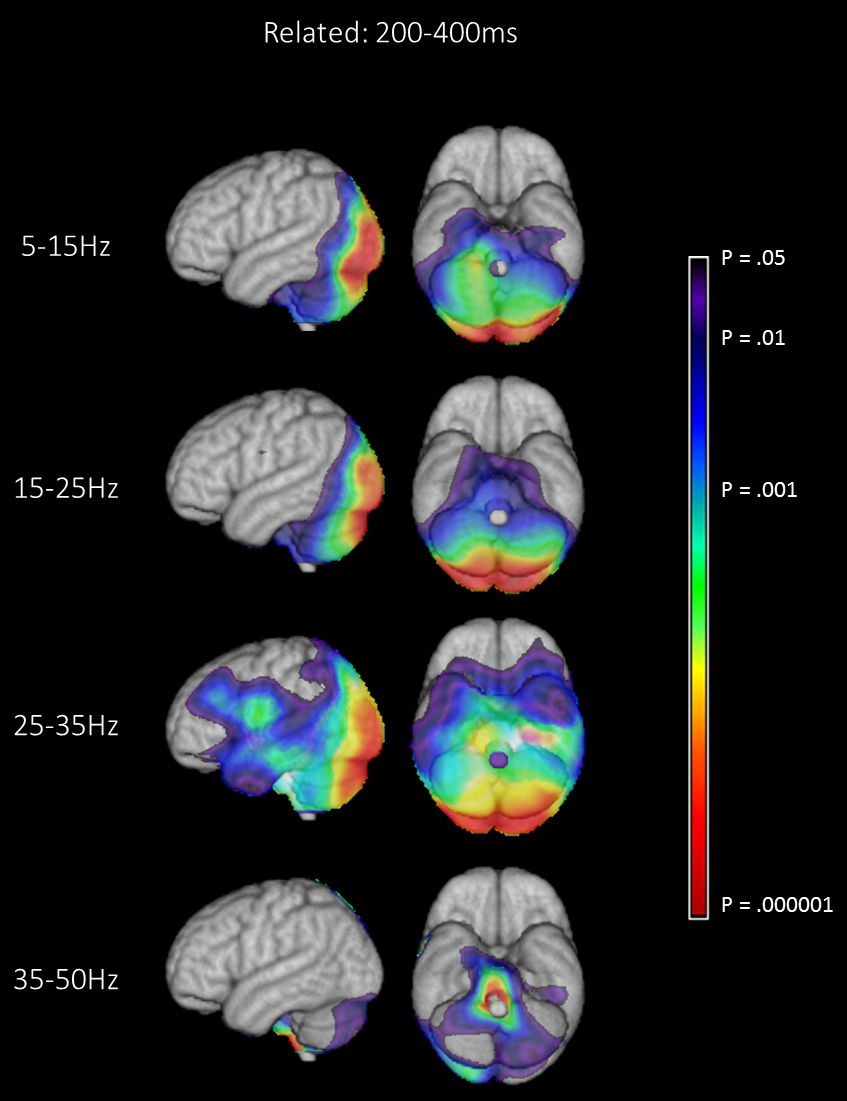


**Supplementary Figure 1:** Additional frequency bands for the 200-400ms time window, related condition

Supplementary Analysis 2: Contrasts between related and unrelated words within the temporal lobe

In addition to the strong/weak differences focused on in the main analysis, we also computed differences between related and unrelated trials to allow comparison with previous studies that employed similar contrasts (for a review, see Lau et al., 2008). The results can be seen in Supplementary Figure 2. In line with this literature, ATL, pMTG and IFG all showed stronger task-related changes in oscillatory power for semantically-associated compared with unrelated items, consistent with the purported role of these sites in semantic retrieval. At ATL and pMTG, there was more sustained engagement for related items at around 10Hz, producing a marked difference between conditions around 400ms post-onset. ATL also showed an earlier response to semantic relatedness around 250ms, from 40-50Hz, demonstrating a relatively rapid sensitivity to semantic variables in ATL, even though the effect of semantic coherence (strong > weak) emerged later, consistent with previous findings (Lau et al., 2013). In pMTG, oscillatory power was also greater for unrelated than related words at 350-400ms at 20Hz. Effects of relatedness were most marked in IFG, which showed both early and late effects, from 0-200 and 400-600ms, centred on 25Hz.

Discussion: The effects of relatedness and strength of association were similar in time-frequency space in ATL – i.e., both effects occurred from 400ms and from 10-15Hz. These effects might reflect a building pattern of coherent semantic retrieval, which is strengthened when related items are highly associated. In pMTG, however, the stronger response to weak associations started soon after the onset of the second word and lasted until 400ms, when the stronger response to related items commenced. If pMTG plays a role in representing a semantic context which can differ from the pattern of long-term associations in ATL, the early and sustained response from 50-400ms could reflect efforts to identify a context in which two weakly-related (or potentially unrelated) words can be coherently associated (while for strong association trials, an appropriate context is already present at the onset of the second word). Finally, in IFG, the early effect of relatedness overlapped with the weak>strong effect at 50ms and 25Hz, suggesting this initial response to meaning reflected controlled retrieval processes. Later, however, IFG showed both effects of relatedness and strong>weak associations: presentation of strong associations expanded the response to related items to include lower frequencies (15Hz to 20Hz. Therefore, we speculate that by 400ms, a pattern of coherent retrieval across semantic sites might be established, eliciting an overall effect of relatedness at all three sites.


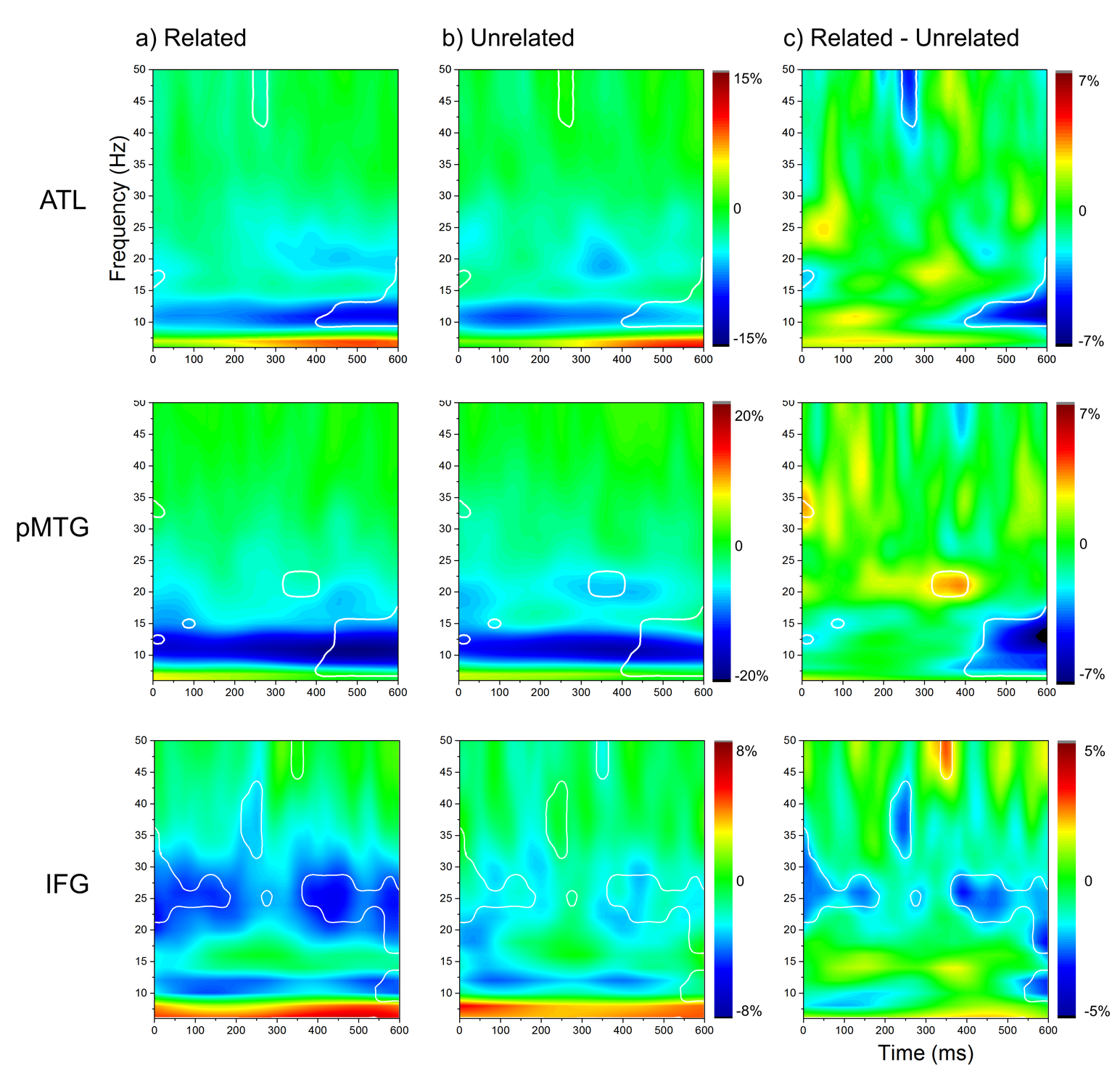


**Supplementary Figure 2:** a) Percentage signal change in the related condition, relative to baseline. b) Percentage signal change in the unrelated condition, relative to baseline. c) Percentage signal change between related and unrelated conditions, separately for ATL and pMTG. White lines are derived from the statistical comparison between related and unrelated conditions. The boundaries enclose regions fulfilling two criteria: i) percentage signal change between the related and unrelated conditions is significantly different from zero (p<0.05) and ii) percentage signal change computed separately for each condition is significantly different from zero for at least one of the two conditions. Yellow-red colours indicate regions of *power increase* relative to the baseline, while cyan-blue indicates *power decreases* relative to the baseline, and green indicates no change from baseline.

Supplementary Analysis 3: Additional TMS analyses

The supplementary analyses below examine (i) the effects of TMS on accuracy for strong and weak associations, and (ii) the effects of TMS on the semantic and number tasks overall. In this analysis, there are 14 as opposed to 15 participants, since data for the even/odd digit task were not recorded for one participant. The results of Analysis 1 motivated the inclusion of accuracy as a covariate in the analysis of response time above. Results from Analysis 2 reveal non-specific effects of TMS and therefore motivated the inclusion of the number control task as a covariate in the main analyses reported above. In addition, Supplementary Table 1 provides full summary statistics for this experiment.

**Supplementary Table 1:** Behavioural data for Experiment 2 (Chronometric TMS)

| **Measurement** | **Site** | **Condition** |  | **0 &**  **40ms** | **125 &**  **165ms** | **250 & 290ms** | **450 &**  **490ms** | **No**  **TMS** |  | **0 &**  **40ms** | **125 &**  **165ms** | **250 &**  **290ms** | **450 &**  **490ms** | **No TMS** |
| --- | --- | --- | --- | --- | --- | --- | --- | --- | --- | --- | --- | --- | --- | --- |
|  |  |  |  | **Mean** | | | | |  | **SD** | | | | |
| RT | ATL | strong |  | 539.53 | 559.05 | 584.54 | 616.85 | 573.43 |  | 120.50 | 138.41 | 156.16 | 195.46 | 143.09 |
|  | ATL | weak |  | 601.16 | 613.62 | 641.42 | 704.06 | 630.98 |  | 167.37 | 174.36 | 192.62 | 214.26 | 163.98 |
|  | ATL | number |  | 535.45 | 565.54 | 576.62 | 616.55 | 571.87 |  | 144.29 | 163.49 | 187.26 | 210.41 | 165.70 |
|  | pMTG | strong |  | 616.55 | 506.63 | 527.08 | 516.72 | 534.54 |  | 210.41 | 132.87 | 145.15 | 130.08 | 119.15 |
|  | pMTG | weak |  | 498.46 | 512.79 | 529.29 | 543.91 | 595.16 |  | 105.49 | 100.93 | 116.69 | 141.97 | 123.20 |
|  | pMTG | number |  | 578.90 | 590.26 | 585.17 | 610.58 | 531.61 |  | 156.50 | 142.16 | 139.25 | 164.37 | 129.47 |
|  | SHAM | strong |  | 510.81 | 499.79 | 531.80 | 550.76 | 551.33 |  | 113.94 | 119.35 | 119.68 | 147.79 | 130.21 |
|  | SHAM | weak |  | 559.64 | 573.55 | 588.73 | 617.25 | 606.62 |  | 133.06 | 144.95 | 145.19 | 158.97 | 125.16 |
|  | SHAM | number |  | 492.95 | 512.32 | 513.29 | 524.02 | 517.83 |  | 126.25 | 132.99 | 127.71 | 145.03 | 127.27 |
| Accuracy | ATL | strong |  | 0.96 | 0.95 | 0.96 | 0.96 | 0.94 |  | 0.04 | 0.06 | 0.04 | 0.05 | 0.05 |
|  | ATL | weak |  | 0.82 | 0.76 | 0.83 | 0.78 | 0.83 |  | 0.13 | 0.17 | 0.11 | 0.18 | 0.08 |
|  | ATL | number |  | 0.92 | 0.92 | 0.95 | 0.95 | 0.92 |  | 0.05 | 0.05 | 0.06 | 0.05 | 0.04 |
|  | pMTG | strong |  | 0.97 | 0.97 | 0.97 | 0.97 | 0.95 |  | 0.05 | 0.03 | 0.04 | 0.03 | 0.06 |
|  | pMTG | weak |  | 0.84 | 0.83 | 0.82 | 0.80 | 0.85 |  | 0.09 | 0.15 | 0.11 | 0.09 | 0.10 |
|  | pMTG | number |  | 0.89 | 0.94 | 0.93 | 0.94 | 0.93 |  | 0.12 | 0.04 | 0.06 | 0.06 | 0.04 |
|  | SHAM | strong |  | 0.97 | 0.97 | 0.97 | 0.97 | 0.95 |  | 0.04 | 0.04 | 0.04 | 0.04 | 0.06 |
|  | SHAM | weak |  | 0.82 | 0.78 | 0.78 | 0.77 | 0.85 |  | 0.11 | 0.09 | 0.15 | 0.14 | 0.11 |
|  | SHAM | number |  | 0.95 | 0.95 | 0.96 | 0.94 | 0.94 |  | 0.07 | 0.04 | 0.05 | 0.05 | 0.05 |

### Effect of TMS on accuracy for strong and weak associations

We wanted to check whether the accuracy of speeded judgements about strong and weak semantic relationships between pairs of words was affected by TMS. To test this, we pooled the total number of correct responses, or hits, for the total number of trials in each block, separately for participant, stimulation site, TMS timing, condition and session. We used these pooled data as input to a generalised linear mixed model (GLMM), implemented in PROC GLIMMIX in SAS v9.4. Since accuracy data have a binomial distribution (hit = 1, 0 = error), we used a logistic link function in the model for the outcome. For the starting model, the fixed effects were: site (ATL, pMTG, sham), TMS time, condition (strong vs. weak) and their two- and three-way interactions. In addition, we included block number and session number as covariates and the per-subject intercept as a random effect. The criteria we used to optimize the final model were: (i) a significant reduction in -2Log-Likelihood between it and the empty model, (ii) only explanatory variables that were statistically significant at p<.05 should be retained. The exception to this was that we had to include the three way interaction between condition (strong vs. weak association), TMS time and site in order to compute post-hoc pairwise comparisons. Supplementary Table 2 shows the outcome for the fixed effects in the final model for which the generalised chi-square per degree of freedom was 1.05, suggesting a good model fit with no over-dispersion. Once the final model was fitted, we used PROC GLIMMIX to estimate pairwise t-tests to compare the LS mean accuracy with and without TMS, carried out separately at each site for each condition. These post-hoc comparisons were controlled for multiple comparisons.

**Supplementary Table 2: Effect of TMS on accuracy for strong and weak associations**

| Model Parameter | | F-value (DF) | p-value | -2Log likelihood |
| --- | --- | --- | --- | --- |
| Empty model  Condition | | 405.40 (1, 1399) | <.001 | 63784.3  63277.5 |
| Condition × Time | | 2.40 (6, 1399) | .026 |  |
| Condition × Time × Site  Block order | | 1.17 (16, 1399)  3.92 (3,1399) | .29  .0084 |  |
|  | |  |  |  |
|  |  | | | |

Plotted separately for ATL and pMTG, Supplementary Figure 3 (upper row) shows the proportion of correct responses for the strong and weak conditions for both the experimental and the sham data. Supplementary Figure 3 (lower row) shows post-hoc paired t-test comparisons between the experimental and sham data, separately for the strong and weak conditions. We found a statistically significant difference between sham and ATL stimulation for weak associations at ~250ms (t(1399) = 2.37, p = 0.018 and between sham and pMTG stimulation for strong associations at ~125ms (t(1399) = 2.08, p = 0.038). These results reflect facilitation of performance, potentially following increases in alertness or motivation (Devlin & Watkins, 2007).

###
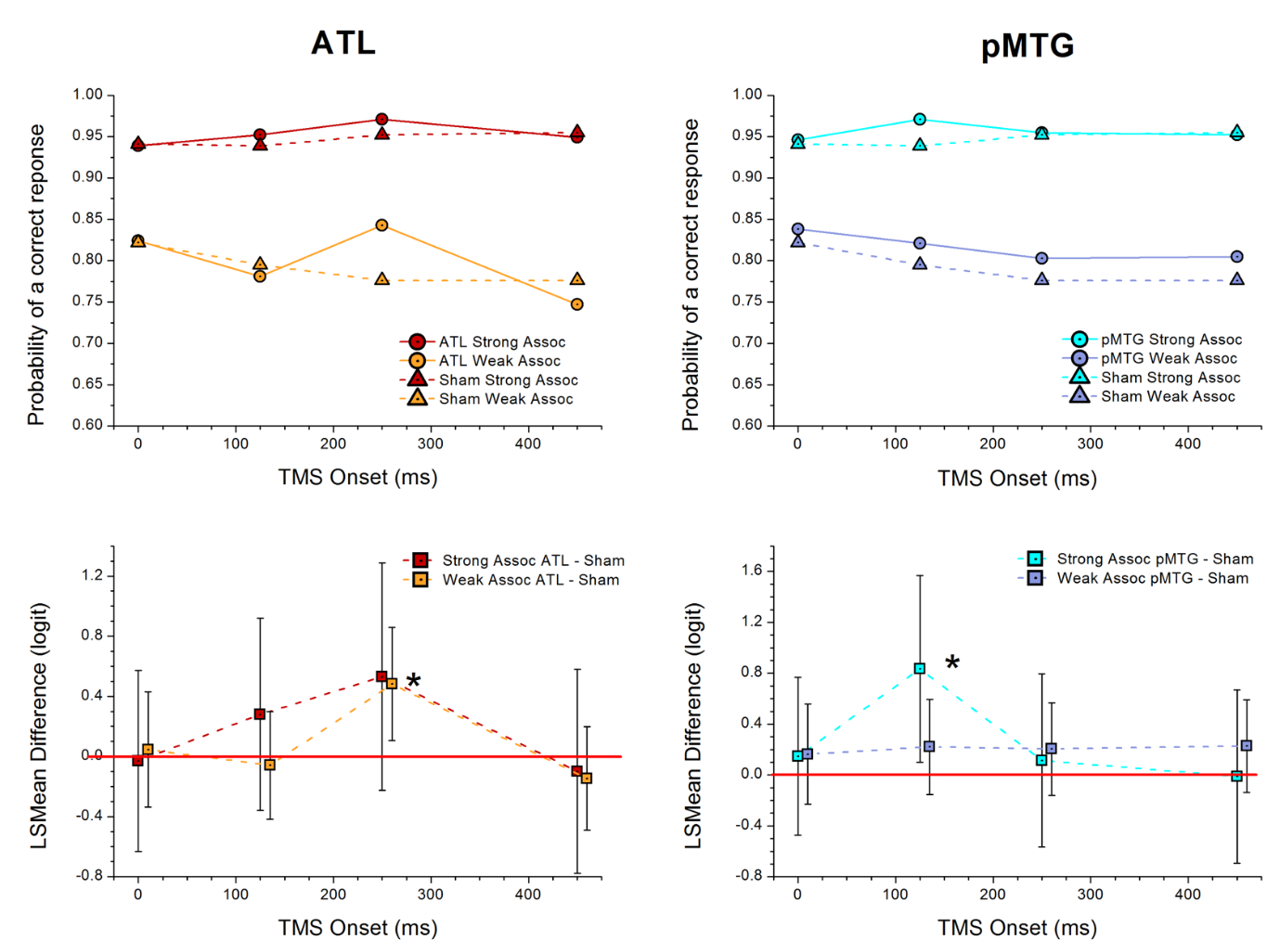


**Supplementary Figure 3**: Effect of TMS on accuracy for strong and weak associations; TOP ROW: Accuracy for strong and weak association for ATL (left) and pMTG (right) relative to sham stimulation. BOTTOM ROW: LS Means difference in accuracy for ATL (left) and pMTG (right) relative to sham site. Points above the red line indicates higher accuracy relative to sham, whereas point below the red line indicates lower accuracy relative to sham. Differences in accuracy (which in all cases correspond to TMS-induced behavioural facilitation) that were statistically significant at p<.05 are indicated by asterisks. Error bars show 95% confidence intervals.

### Effect of TMS on response times in the semantic word vs. number control task

We wanted to test whether there might be non-specific effects of administering chronometric TMS that should be controlled for in the main analysis of reaction times in relation to strong versus weal semantic association. To do this we compared reaction times in the digit parity task with the semantic task overall. We used PROC MIXED in SAS v9.4 to build an initial GLMM with the following fixed effects: site (ATL, pMTG, sham), TMS time, condition (semantic vs. number task) and their two- and three-way interactions. The starting model also included block number, session number and mean accuracy per block as covariates. We included the per-subject intercept as a random effect. Incorrect responses and outlying data points that fell more than 2 SD from each participant’s mean RT were removed, for each session, prior to analysis. The criteria we used to optimize the final model were: (i) a significant reduction in -2Log-Likelihood between it and the empty model, (ii) only explanatory variables that were statistically significant at p<.05 should be retained. Once the final model was fitted, we used PROC MIXED to estimate pairwise t-test comparisons between experiment and sham reaction times at each time point for TMS administration, computed separately for ATL and pMTG as well as word versus number stimuli. All post-hoc comparisons were controlled for multiple comparisons. The fixed effects that were retained in the optimized model are reported in Supplementary Table 3. We found statistically significant main effects of TMS time, condition (number vs. word task) and site (ATL vs. pMTG vs. sham), and we retained only the non-significant three-way interaction in order that we could compute the pair-wise post-hoc comparisons. The covariates of block and session order were also statistically significant at p<.05.

**Supplementary Table 3: Effect of TMS on RT to semantic and number parity tasks**

| Model Parameter | F-value (DF) | Z-value | p-value | -2Log likelihood |
| --- | --- | --- | --- | --- |
| Empty model  Time | 53.80 (3, 11000) |  | <.001 | 146110.3  144796.6 |
| Site | 122.78 (2, 11000) |  | <.001 |  |
| Condition × Time × Site  Block order | 1.20 (17, 11000)  41.10 (3, 11000) |  | .26  <.001 |  |
| Testing session | 178.80 (2, 11000) |  | <.001 |  |

| Participant covariance |  |  | 2.74 | .0031 |  |  |
| --- | --- | --- | --- | --- | --- | --- |

As illustrated in Supplementary Figure 4, post-hoc comparisons for the semantic task showed statistically significant differences between LS mean RT for ATL stimulation compared to sham at all four time points (0ms: t(11000) = 4.38, p <.001; 125ms: t(11000) = 5.72, p <.001; 250ms: t(11000) = 5.38, p <.001; 450ms: t(11000) = 6.90, p <.001). We found no equivalent differences between sham and pMTG stimulation. Post-hoc comparisons for the number task showed statistically significant differences in LS mean RT for the ATL stimulation compared to sham at all four time points (0ms: t(11000) = 4.50, p <.001; 125ms: t(11000) = 4.10, p <.001; 250ms: t(11000) = 5.25, p <.001; 450ms: t(11000) = 6.97, p <.001), as well as for pMTG relative to sham at 450ms (t(11000) = 3.25, p = 0.0012). These results suggest that despite the use of a staircase procedure (Sliwinska et al., 2012) which was designed to reduce sensitivity to the variability in TMS onset time (see Methods), RTs increased with systematically with TMS onset time. This may correspond to an expectancy effect in participants who waited for the TMS pulse to have occurred before responding. This interpretation is made all the more plausible because we found a similar pattern of increasing RTs following sham stimulation which was administered at 30% of stimulator output. This is not thought to be strong enough to stimulate cortex (Duecker et al., 2013) but is sufficient to produce scalp sensations. The increase in RT, relative to sham, was more marked for ATL than for pMTG, which may reflect the stronger scalp sensations associated with ATL stimulation.

**
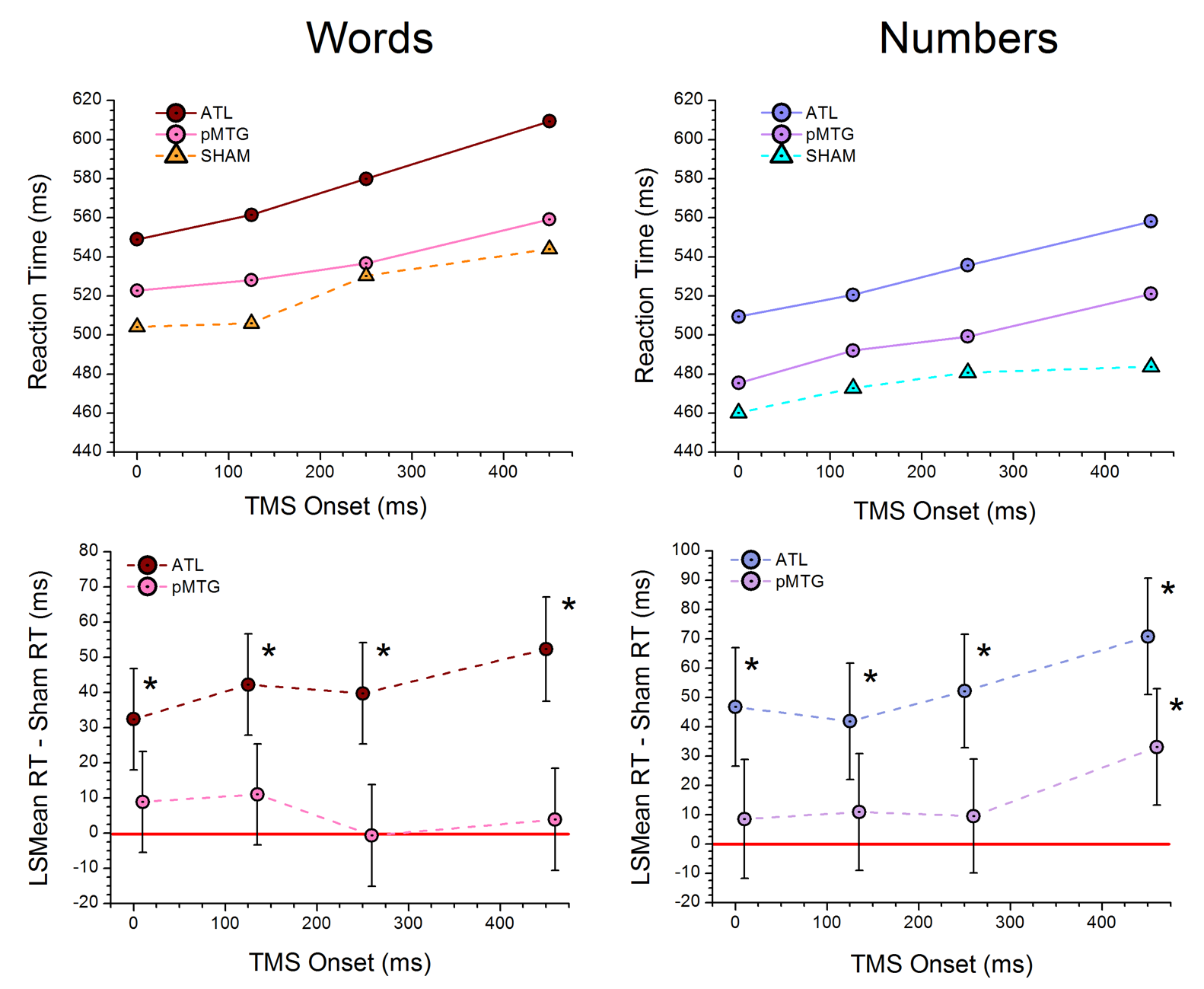
**

**Supplementary Figure 5:** Effect of TMS on RT for semantic and digit parity tasks; TOP ROW: RT (ms) for three sites for semantic (left) and digit task (right). BOTTOM ROW: LS Means difference in RT for ATL and pMTG relative to sham site for semantic (left) and digit (right) task. Points above the red line indicate longer RT relative to sham, whereas points below the red line indicate faster RT relative to sham. Differences which are statistically significant at p<.05 are indicated by asterisks. Error bars show 95% confidence intervals.
